# When Stents Go Astray, We Find a Way: A Case Report on Retrieving a Migrated Esophageal Stent

**DOI:** 10.7759/cureus.67009

**Published:** 2024-08-16

**Authors:** Vinayak Venu, Girish Bakhshi, Aishwarya Dutt, Apoorva Raichur, Naman Jaiswal

**Affiliations:** 1 General Surgery, Grant Government Medical College and Sir JJ Group of Hospitals, Mumbai, IND

**Keywords:** gastroesophageal disease, gastroesophageal surgery, esophagus and gastric cancer surgery, corrosive injury of the esophagus, upper endoscopy, esophageal sems, lumen-apposing self-expanding metal stents, esophageal stenosis, general surgery

## Abstract

Benign esophageal strictures are characterized by the narrowing of the esophageal passage due to fibrotic changes. These strictures can arise from various causes, including gastroesophageal reflux disease, which leads to peptic strictures; surgical procedures causing esophageal injury, resulting in anastomotic strictures; radiation therapy, ingestion of corrosive substances, or endoscopic resection. Approximately 10% of benign esophageal strictures do not respond to conventional dilation therapy, prompting the consideration of temporary stent insertion as an alternative treatment approach. However, only about one-third of patients with refractory benign esophageal strictures experience sustained relief from dysphagia following self-expanding stent placement. Challenges such as stent migration and hyperplastic tissue response pose limitations to the effectiveness of this intervention.

The utilization of self-expanding metal stents (SEMSs) in benign esophageal diseases is not standard practice due to the associated risks of adverse events such as tissue ingrowth at the uncovered portions, migration, and bleeding. One of the major challenges encountered is the growth of hyperplastic tissue around the stent during retrieval and subsequent serial esophageal bougie dilations. Long-term self-bougie dilations, coupled with the patient's gained self-confidence, played a crucial role in the management. While most migrated esophageal metallic stents are typically left in the stomach, in this particular case, the patient's progressive dysphagia necessitated retrieval.

This article discusses a 65-year-old female with a benign esophageal stricture treated with a self-expandable metallic stent. Eight months post-insertion by another doctor, she presented to us with worsening dysphagia. Endoscopy revealed a stent migrated into the antrum of the stomach with a proximal esophageal stricture. Endoscopic dilation and stent retrieval were performed, followed by serial esophageal bougie dilations. Subsequently, her dysphagia settled with self-insertion of a 9 mm esophageal dilator.

## Introduction

Benign esophageal stricture (BES), characterized by the fibrotic narrowing of the esophageal lumen leading to dysphagia, is a common finding in routine endoscopic examinations. The development of esophageal stricture is initiated by inflammatory processes, followed by proliferation and remodeling, resulting in fibrotic changes and scar formation [1]. Furthermore, biomechanical degradation and alterations in collagen components within the submucosal layer contribute to the pathogenesis of esophageal stricture [2]. Despite these known factors, the molecular mechanisms underlying esophageal stricture remain largely unexplored, primarily due to the absence of a reliable animal model for studying this condition. Common causes of benign esophageal stricture include gastroesophageal reflux disease, surgical injury (anastomotic), radiation therapy, ingestion of caustic substances, or endoscopic resection or ablation [3].

The primary therapeutic approach for managing BES is endoscopic dilation (ED), typically conducted through stepwise bougie or balloon dilations [4]. In around two-thirds of patients, satisfactory dilation is achieved within three to five ED sessions [5]. However, in the remaining one-third of patients, refractory BES may develop, necessitating more ED sessions to alleviate dysphagia. Given the demanding nature of ED for patients and its associated hospital and treatment costs, alternative treatment options have emerged to either circumvent the need for ED or reduce the frequency of ED sessions during follow-up. These options include needle-knife stricture incision for anastomotic strictures, stricture injection with corticosteroids, or the placement of a self-expandable metal or biodegradable stent [3,6,7]. Refractory or recurrent benign esophageal strictures occur when there is either an inability to maintain a stricture to a diameter of 14 mm over 5 sessions at 2-week intervals (refractory) or an inability to sustain a satisfactory luminal diameter for 4 weeks after achieving a diameter of 14 mm (recurrent) [8].

## Case presentation

A 65-year-old woman with a chronic, refractory benign gastroesophageal junction stricture, who had previously undergone multiple esophageal dilatations, received a fully covered self-expandable metal stent (18 mm x 123 mm) inserted 8 months ago at another institution. She presented to our Outpatient Department with progressive dysphagia for solid foods. Despite experiencing dysphagia, the patient did not follow up for upper endoscopy or subsequent appointments after the stent insertion. Eight months after the insertion of the metallic stent, the patient's dysphagia worsened, limiting her to a liquid diet and prompting her to seek medical assistance. During gastroscopy, an esophageal stricture was identified, along with the fully embedded previously placed self-expandable metal stent (SEMS), which could not be manipulated via the endoscope.

**Figure 1:**
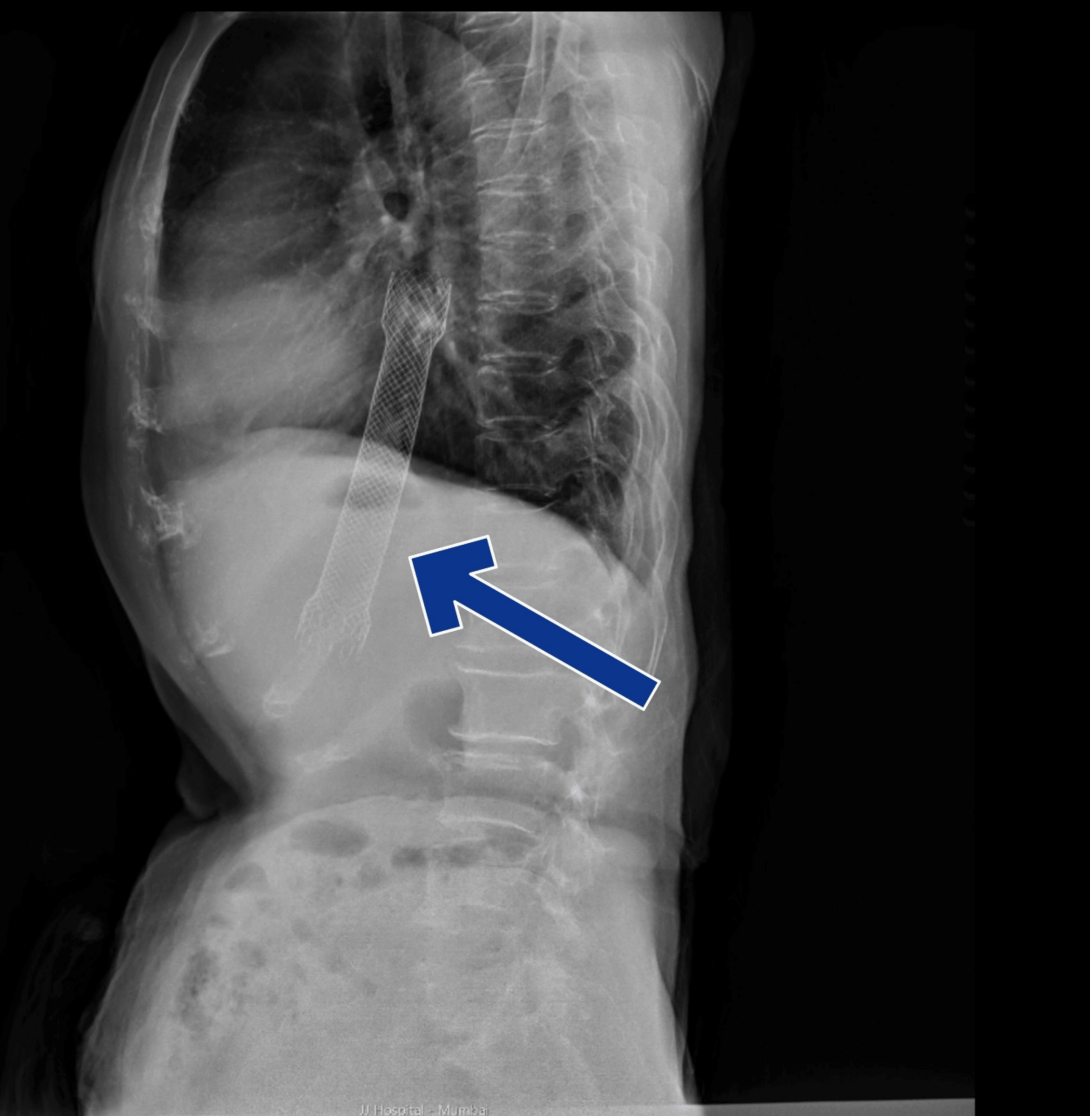
X-ray lateral view of thorax showing the distally migrated metallic stent (blue arrow)

**Figure 2:**
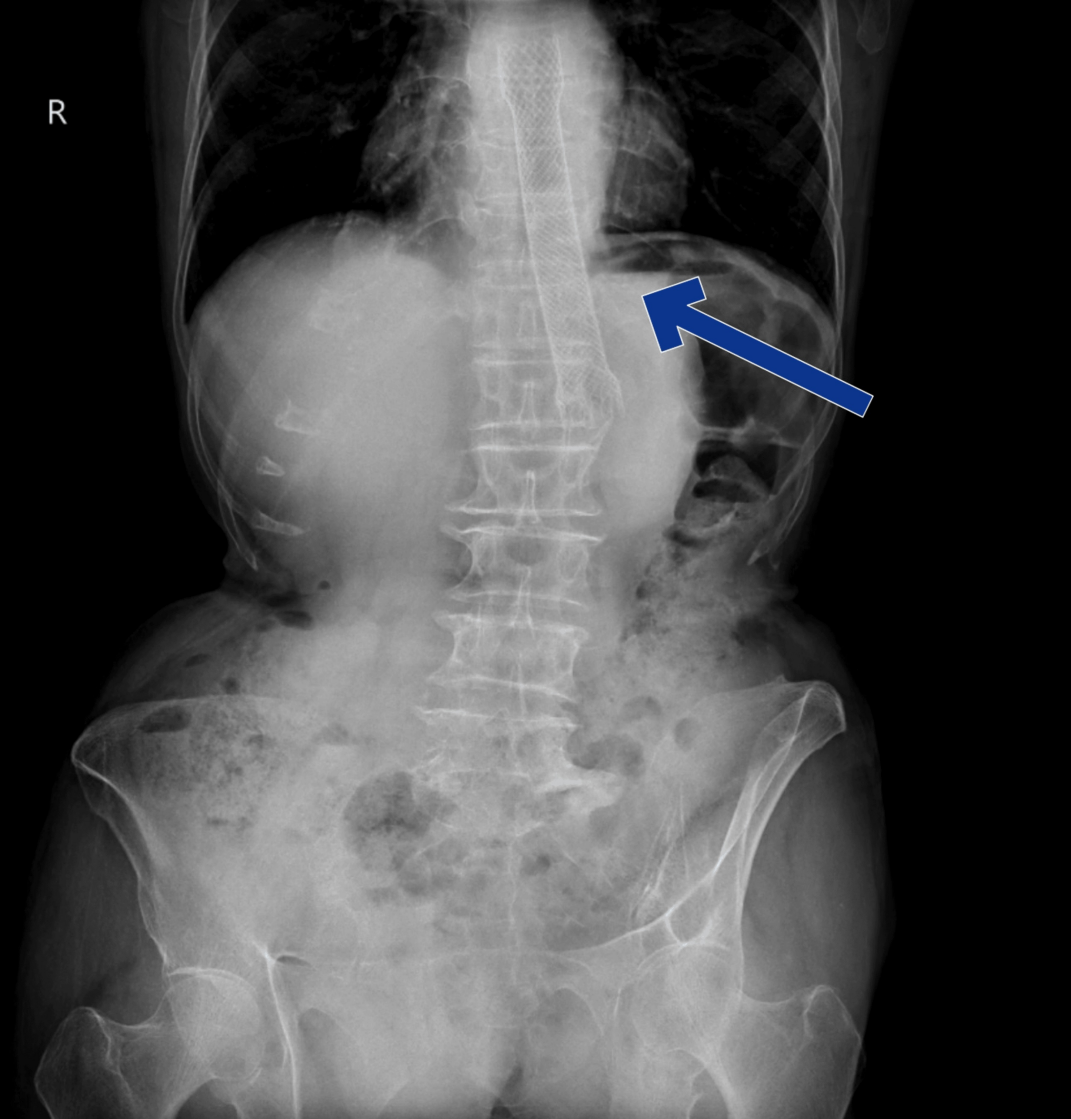
X-ray posterior-anterior view of the thorax showing the distally migrated metallic stent (blue arrow)

At the time of presentation, the patient was hemodynamically stable and tolerating a liquid diet. Initial X-rays of the abdomen and chest revealed a distally migrated metallic stent (Figure 1, Figure 2).

**Figure 3:**
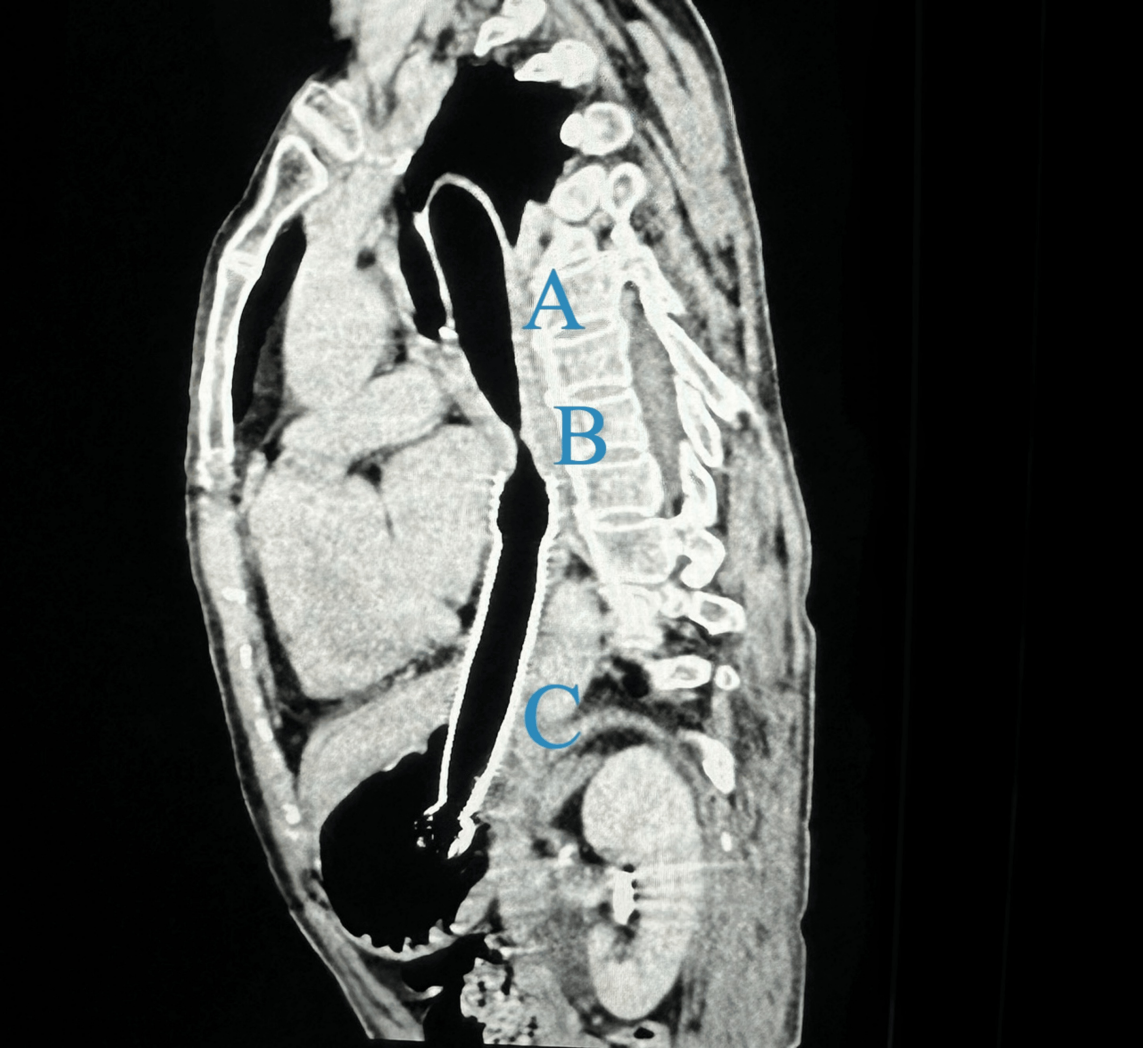
Computed tomography saggital section of the thorax showing: A- dilated proximal part of the esophagus, B- esophageal stricture with a residual diameter of 3 mm from the D6 to D8 levels, C- esophageal stent seen with the distal end in the gastric antrum

Computed tomography of the thorax and abdomen revealed an esophageal stricture with proximal esophageal dilation. Furthermore, it showed a migrated stent extending distally into the gastric antrum (Figure 3).

**Figure 4:**
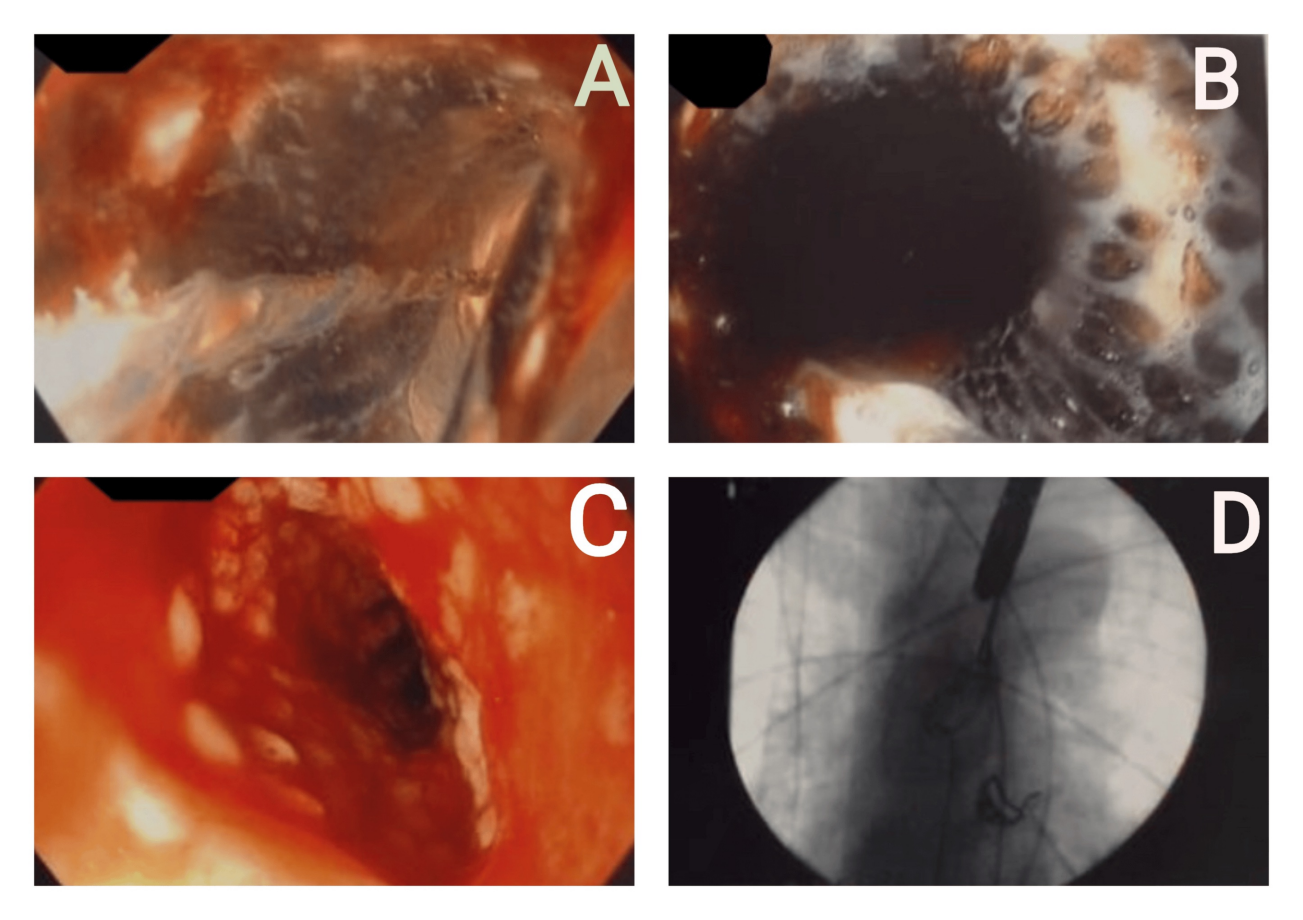
A: Endoscopic view showing an esophageal stricture at a distance of 30 cm from the incisor. B: Endoscopic view showing the proximal end of the metallic stent. C: Friable mucosa after stent removal with no evidence of bleeding or growth. D: Fluoroscopic view confirming complete retrieval of the metallic stent.

The patient underwent endoscopic dilatation of the esophageal stricture up to 12 mm and retrieval of the metallic stent using forceps. The residual esophageal mucosa was found to be friable, with no evidence of malignant growth (Figure 4).

The patient's dysphagia improved with tolerance to a solid diet. The next step in treatment was to maintain the residual diameter of the esophagus through progressive esophageal dilation. The patient underwent serial esophageal bougie dilation, starting from a 5 mm internal diameter, with assistance from the surgeon. The patient was guided in the self-insertion of an esophageal bougie and successfully conducted consecutive esophageal bougie dilations independently. Emotional support from counseling and family members played a crucial role throughout the process. After five days of consecutive self-bougie dilation, the patient achieved successful self-insertion of an esophageal dilator with a 9 mm internal diameter. Subsequently, the patient was discharged on a solid diet with a customized esophageal self-dilator bougie with a 9 mm diameter. Currently, the patient is performing self-esophageal dilation using a 9 mm diameter self-bougie esophageal dilator daily before breakfast. Additionally, the patient is on regular monthly follow-up appointments to monitor progress and ensure optimal management of the condition.

## Discussion

Fully covered self-expandable metal stents (fcSEMS) are favored for the treatment of refractory benign esophageal strictures. However, a significant drawback of fcSEMS is their relatively high risk of migration. Previous studies, including a meta-analysis of 18 studies involving 444 patients, reported stent migration in 29% of patients with benign strictures [9]. In certain clinical scenarios, stent migration may be a favorable outcome, indicating the resolution of benign strictures or a positive response of malignant strictures to treatments like chemoradiation therapy [10].

While various methods have been proposed to address stent migration and its management, this issue persists as a challenge. If a self-expandable metal stent migrates to the stomach, its removal is warranted due to the potential risks of gastrointestinal obstruction and perforation. Although migrated stents are frequently found within the esophagus and can typically be repositioned or extracted through endoscopic procedures, addressing a stent that is lodged distally to an esophageal stricture presents a unique dilemma.

Self-dilation for benign esophageal strictures typically occurs toward the conclusion of the treatment algorithm. This approach is often overlooked or not selected due to limited experience in teaching the technique. The objective is to achieve a target bougie size of 14 mm, enabling patients to consume solid foods [4]. Complications are infrequent when patients adhere to a well-structured training program. Ideally, this technique is suitable for simple, proximally located strictures according to current literature. However, there is no specific protocol designed for the frequency of self-dilation in the literature according to the target bougie size.

The University of Michigan study demonstrated that after a median duration of nearly 10 years, 47% of patients with a cervical esophagogastric anastomotic stricture had ceased self-dilation while the remaining 53% continued self-bougienage at an average frequency of once every 2 months [11]. In contrast, in the series by Dzeletovic et al., which included a more diverse patient population, 10% of patients were able to discontinue self-dilation, and 27% had reduced the frequency of self-bougienage to a maximum of twice weekly [12]. These findings suggest that once a satisfactory bougie size is achieved, the frequency of self-bougienage can be gradually decreased, thereby further alleviating the burden on the patient. In the patient discussed in this case report, the achieved bougie size was only 9 mm, thereby limiting her to self-dilate daily for the first month, followed by twice a week thereafter.

A retrospective analysis was conducted on 51 patients with corrosive esophageal strictures treated in an Indian surgical unit [13]. Patients underwent instruction in self-dilatation using gum elastic bougies as the final treatment step, participating in a progressive domiciliary program. Quarterly follow-ups over one year ensured adherence to the self-bougienage technique. Treatment outcomes were uniformly positive, with all patients experiencing significant relief from dysphagia and demonstrating improved health and barium study results. Six patients encountered mediastinitis during initial dilatation, all of whom recovered with conservative care. Only one patient required readmission due to non-compliance with self-bougienage, but after re-training, remained symptom-free.

## Conclusions

This case report discusses the treatment of a 65-year-old woman with a chronic, refractory benign gastroesophageal junction stricture who received a self-expandable metallic stent. Despite the stent migrating to the gastric antrum and causing dysphagia, successful endoscopic retrieval, followed by serial esophageal bougie dilations, enabled the patient to self-insert an esophageal dilator, leading to improved dysphagia and discharge on a solid diet.

While self-dilation for benign esophageal strictures typically occurs toward the end of the treatment algorithm, the frequency of self-bougienage varies based on individual patient characteristics and the achieved bougie size. Studies have shown that once a satisfactory bougie size is attained, the frequency of self-dilation can be gradually reduced, further alleviating the burden on the patient. There is a critical need for a multidisciplinary approach in managing migrated esophageal stents with strictures, encompassing procedures, such as esophageal retrieval and multiple dilations, with a significant emphasis on the patient's self-confidence throughout the treatment process. Further research is needed to establish standardized protocols regarding the frequency of self-dilation, considering the bougie size, and the management of stent migration distal to the stricture.
